# Community psychiatric care for people with mental disorder and homelessness, with the involvement of peer support. Cooperation of the Awakenings Foundation and BMSZKI

**DOI:** 10.1192/j.eurpsy.2024.278

**Published:** 2024-08-27

**Authors:** T. Bulyáki, J. Harangozó, Z. Harangozó, P. Kéri

**Affiliations:** ^1^Department of Social Work, ELTE TÁTK; ^2^Community Psychiatric Center-Awakenings Foundation, Semmelweis University; ^3^BMSZKI; ^4^GAMIAN-Europe; ^5^Awakenings Foundation, Budapest, Hungary

## Abstract

**Introduction:**

A person diagnosed with a psychiatric illness, must face labels and discriminiation most of the time. Fear of these undermines the motivation of people in need to seek help. A special example of this phenomenon is the case of people experiencing homelessness and mental disorder, avoiding the additional stigma of homelessness and therefore do not seek any help for their mental ill-health. Availability of the specific services complicates their problem.

Fear of stigma, trauma, and previous bad experiences of using services also keep people experiencing homelessness away from services.

In Hungary, the February Third Working Group (F3) Report on the 2020 Homelessness Survey After the Penal Code - Before the Pandemic Homelessness - Services Perspectives by Péter Győri shows in his summary paper that only 29% had received psychiatric treatment.

**Objectives:**

Methodology Center of Social and Its Institutions (BMSZKI), in collaboration with the Awakenings Foundation, developed a complex rehabilitation service for people experiencing homelessness and mental disorder. This presentation aims to present this good practice.

**Methods:**

Complex rehabilitation based on the methodology of community psychiatric care with the involvement of peer support.

**Results:**

- provision of community psychiatric care for people experiencing homelessness and mental disorder,
- introduction of screening for effective care of undiagnosed persons with mental disorders,
- provision of outpatient and day hospital care
- focus of care in accommodation services on persons with mental disorders,
- the involvement of peer-support work in the service,
- building a network of contact points, organizing case conferences,
- developing and organizing training on recovery-based rehabilitation for people with mental disorders in cooperation between the two organizations,
- telemedicine, making digital mental health available
- presence of resources represented by self-help groups
- running a working group to promote improvements based on practical experience homelessness and mental disorder.

**Conclusions:**

extra-institutional teamwork multiplies the resources for people experiencing homelessness and mental disorder.

**Keywords:**

mental disorder, homelessness, community psychiatric care, peer support, collaboration

**Disclosure of Interest:**

None Declared

